# The Strategies to Homogenize PET/CT Metrics: The Case of Onco-Haematological Clinical Trials

**DOI:** 10.3390/biomedicines4040026

**Published:** 2016-11-15

**Authors:** Stephane Chauvie, Fabrizio Bergesio

**Affiliations:** Medical Physics Unit, Santa Croce e Carle Hospital, Cuneo 12100, Italy; fabergesio@gmail.com

**Keywords:** positron emission tomography, standardization, clinical trials, biomarker, standardized uptake value, lymphoma

## Abstract

Positron emission tomography (PET) has been a widely used tool in oncology for staging lymphomas for a long time. Recently, several large clinical trials demonstrated its utility in therapy management during treatment, paving the way to personalized medicine. In doing so, the traditional way of reporting PET based on the extent of disease has been complemented by a discrete scale that takes in account tumour metabolism. However, due to several technical, physical and biological limitations in the use of PET uptake as a biomarker, stringent rules have been used in clinical trials to reduce the errors in its evaluation. Within this manuscript we will describe shortly the evolution in PET reporting, examine the main errors in uptake measurement, and analyse which strategy the clinical trials applied to reduce them.

## 1. Introduction

Cancer is now the second most common cause of death immediately after cardiovascular acute events. For a number of many common cancers, treatment of disseminated disease is often non-curative, toxic, and costly. Due to the extreme variability of tumour response to antineoplastic treatment of different cancers, static and functional imaging plays an essential role in antineoplastic treatment efficacy assessment.

The [18F]-fluoro-2-deoxy-d-glucose (FDG) positron emission tomography computed tomography (PET/CT) has nowadays assumed a pivotal role in oncology for tumour’s staging, restaging and follow-up, thereby paving the way to personalized medicine [1,2].

PET/CT has already been a fundamental tool for staging and restaging in lymphoma [3,4] and its use is now standardized in the so-called Lugano criteria [5]. Recently, several clinical trials demonstrated that treatment could be guided by PET/CT also during the treatment itself, changing the ongoing therapy based on its results [6,7]. The research in this field is still ongoing and requires a lot of effort, but PET/CT became a true tool for personalized medicine when its results started to be given on a discrete scale [8,9]. Indeed, PET/CT has always been interpreted in a binary way, identifying the areas of high uptake and comparing them with the surrounding background [10]. However, if PET/CT is used for therapy assessment by identifying the change in tumour uptake, the use of a discrete scale in which the tumour lesion is compared to reference tissues and organs is needed. Furthermore, the idea of quantitative assessment of the uptake in PET/CT scan has been introduced in literature [11] upgrading the discrete scale to a full continuous scale, a PET/CT biomarker able to detect whichever variations are present in the uptake. In addition, standardized uptake value (SUV), metabolic tumour volume (MTV) and total glycolytic volume (TGV) have been proposed as gauges of tumour burden and aggressiveness [12] and are valid candidates for this scale.

Before being implemented in clinical practice, the new assessment tools ought to be proven in the controlled and reproducible environment of clinical trials. Indeed, both the discrete and the continuous scale depend on PET/CT uptake, which is affected by several confounding factors. Within this review we will discuss briefly which are the major sources of errors affecting the image reading and we will show how they were managed within several onco-haematological clinical trials. In particular we will analyse the three strategies that shall be done to assure the highest reproducibility of the results: the use of a central review for PET/CT assessment, the equalization of PET/CT scanner, and the harmonization of PET/CT procedure.

## 2. Analysis and Reporting of Positron Emission Tomography Computed Tomography (PET/CT) Images

As previously mentioned, PET/CT reporting has always been based more on the extent of the disease (identifying the different tumour foci) more than on the avidity of the tumour itself, even if it is well known that the uptake of FDG is correlated to the cell metabolic rate [13]. The description of the tumour avidity could be done in three different scales: binary, discrete and continuous.

### 2.1. Binary Scale

The areas of uptake are defined as present if their uptake is higher compared to the surrounding background. Difficulties in interpretation are encountered when tumours are located in areas of high uptake or when the variations between different scans of the same patients are small (i.e., during treatment assessment). It is easy to perform in clinical settings and it has no stringent requirements on acquisition. It could be done with standard-of-care software usually available in off-the-shelf workstations.

### 2.2. Discrete Scale

**Figure 1:**
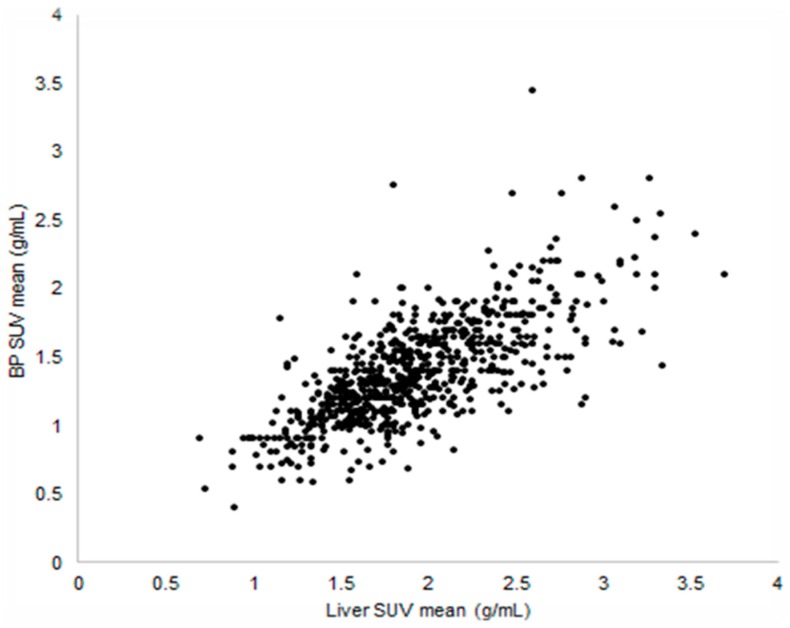
SUV (standardized uptake value) mean in the blood pool versus liver in a cohort of 1132 patients acquired on 56 different PET/CT scanners.

The areas of uptake are compared to normal, disease-free organs and tissues as an internal scale. The most commonly used references are the liver and the blood pool, as in Deauville [8] and in PET Response Criteria in Solid Tumors (PERCIST) [9] criteria. The liver and the blood pool, usually measured in a large vessel such as the aorta, are indeed very stable [14] if images are acquired in a steady state, starting the acquisition at least 45 min after the FDG administration. In Figure 1, we can see how the uptake in the liver and in the blood pool in a large sample of PET/CT scans is nearly constant, demonstrating that these uptakes could be used as an internal scale. The clear advantage of using a discrete scale for rating tumour avidity is that it could be used both for staging and for restaging, even when the variations between different scans of the same patients are small (i.e., during treatment assessment). The application of a discrete scale requires little training for the readers, and an excellent inter-reader variability was demonstrated in the literature for Hodgkin lymphoma (HL) [15,16,17] and non-Hodgkin lymphoma (NHL) [18,19]. Moreover, it is easy to perform in clinical settings, could be done with standard-of-care software, and only requires that uptake of different scans of the same patient remain inside a tolerable limit. On the other hand, it does not overcome completely the problem of visual analysis: two areas with the same uptake could be misclassified if they are surrounded by a different background. We know indeed that the eye overestimates the uptake in an area surrounded by a low background in comparison to the same area surrounded by a high background [20]. Some authors proposed using SUV measurements in those areas to override this problem [19], but this has not been studied extensively and actually moves the classification of the PET analysis from a discrete to a continuous scale with the issues described in next section.

### 2.3. Continuous Scale

The areas of uptake, once identified, are measured by associating a continuous variable, which gives the size of the uptake, and a unit describing which physical entities we are measuring. The different PET-derived metrics, SUV, MTV and TGV are described in the following sub-chapters with their strengths and limitations.

#### 2.3.1. Standardized Uptake Value

SUV is the semi-quantitative PET metric most frequently used for measuring tumour glucose metabolism. It is defined as the ratio of the decay-corrected concentration of activity ([A_tissue_]) in the tissue to the injected activity (A) normalized to the patient’s body weight (BW):

(1) $$SUV\;\left(\frac{g}{mL}\right)=\frac{\left[A_{tissue}\right]\left(\frac{Bq}{mL}\right)\times BW\left(g\right)}{A\left(Bq\right)}$$

To account for the different bio-distribution of FDG in tissue and fat, SUV corrected for lean body mass (SUL) has been introduced recently [9] and it is defined by the following formulas:

(2) $$LBM_{male}=1.1BW-120{\left(\frac{BW}{Height}\right)}^{2}$$

(3) $$LBM_{female}=1.07BW-148{\left(\frac{BW}{Height}\right)}^{2}$$

Even if SUL has not been yet used extensively in clinical practice, the general advice, furnished by some guidelines [21,22], is to collect SUL along with SUV data to further understand its relevance both in clinical practice and for experimental settings.

Semi-quantitative metrics are calculated inside a volume of interest (VOI) which is drawn on PET images and that is representative of the tissue being analysed. Depending on the VOI delineation, a few simple SUV-based metrics are defined:

- *SUV*_max_: it is the maximum value within the VOI; this index is simple to measure and provides information about the most active tumour foci. Its drawback is the strong dependence on image noise, because it corresponds to a single voxel measure.
- *SUV*_mean_: it is the average of all values belonging to the VOI; this metric evaluates the mean metabolic activity of tumour. It is much less vulnerable to image noise, but heavily depends on the delineation method used for drawing the VOI [23].
- *SUV*_peak_: this value represents the maximum tumour activity within a 1 cm^3^ VOI in the hottest part of the tumour volume [22,23]. The rationale is to have an index measurement associated with the hottest part of the tumour, but in a standard volume, less dependent on noise.

#### 2.3.2. Metabolic Volumes

Recently, two other metrics have been defined to characterize the tumour burden: they are called metabolic tumour volume (MTV) and total glycolitic volume (TGV), also indicated as total lesion glycolysis (TLG).

MTV measures the total volume of metabolically active tumour included within a VOI and is expressed in cm^3^ or mL. This index evaluates the extent of disease and it is based on the assumption of a metabolic activity higher than the surrounding healthy tissue to be able to accurately define the tumour volume. MTV is less affected by noise since it includes hundreds or thousands of voxels.

TGV is defined by the following relation:

(4) $$TGV\left(g\right)=SUV_{mean}\times MTV$$

The rationale is to combine tumour burden (volume) and its metabolic activity (uptake). This metric evaluates tumour aggressiveness.

Metabolic tumour volume measurements are of crucial importance to pursue a quantitative approach to PET: to the state of the art, MTV and TGV do not have the three qualities of precision, accuracy and repeatability, which are requisites of a good prognostic index.

#### 2.3.3. Kinetic Modelling

Moreover, the most accurate metric—kinetic modelling that describes the delivery, retention and utilization of glucose—is difficult to perform since it requires long dynamic PET scans, personnel with specific knowledge and dedicated software for the analysis. It is usually used in single-centre trials to perform pharmacodynamics studies, in phase I–II studies or in novel non-FDG tracers.

## 3. Sources of Errors in Uptake Evaluation

The sources of error in uptake evaluation can be divided into three groups, according to the dependence on the PET/CT scanners, the site procedure for patient preparation and acquisition, and on the patient itself. Valid recommendations have been released by the US and European nuclear medicine associations [21,22] in order to provide minimal standards. The general prescription is to reach a high level of standardization: this aspect is essential to guarantee an efficient comparison of PET/CT metrics acquired at different time points (intra-patient) and between different patients (inter-patient), either at a single site or across multiple sites [24,25]. In the next paragraphs we will describe shortly which are the major sources of errors in uptake evaluation that should be taken in account while planning PET/CT-oriented clinical trials. We will also inform the reader of the aforementioned guidelines and to specific reviews [26,27] for the detailed description of all the factors affecting uptake evaluation.

### 3.1. Scanner-Related Factors

These factors depend on the equipment used by the site: the PET/CT scanner to acquire the images of the patient and the dose calibrator used for measuring the FDG activity. The scanner-related factors are usually addressed, within a clinical trial, by a central imaging corelab.

#### 3.1.1. Cross Calibration of PET/CT Scanners

Cross-calibration of PET scanners and dose calibrators are mandatory to minimize uptake variability. Cross-calibration consists in the acquisition, as detailed by the PET/CT manufacturer’s manual, of a cylindrical phantom in which an activity of ^18^F, measured with the dose calibrator, has been inserted. A calibration factor is set on the PET/CT scanner in such a way that the activity measured by the scanner is the same measured by the dose calibrator. Since the dose calibrators are usually calibrated to 3%–10% precision and there could be some errors in the filling procedure of the phantom, the accepted difference in clinical practice between the activities measured by the scanner and the dose calibrator is 10%. It is advisable to always use the same dose calibrator to measure the activity employed for calibrating the scanner. If more than one calibrator are used, they should all be cross-calibrated with a traceable radioactive source. Indeed, the injected activity into the patient must always be measured with the calibrator, which has been cross-calibrated with the PET/CT scanner used for imaging. PET/CT sites not equipped with dose calibrators cannot get reliable uptake measures.

Variability among different scanners, even in a controlled environment of a multi-centre clinical trial, proved to be up to 25% [28]. Effort of cross-calibration through the measurements of different phantoms permits achievement of a variability less than 10% in multi-centre clinical trials [23,24,25,26,27,28,29,30], while 5% should be a good requirement for using PET/CT in a quantitative way [31,32].

#### 3.1.2. Verification of Image Reconstruction Algorithm

**Figure 2:**
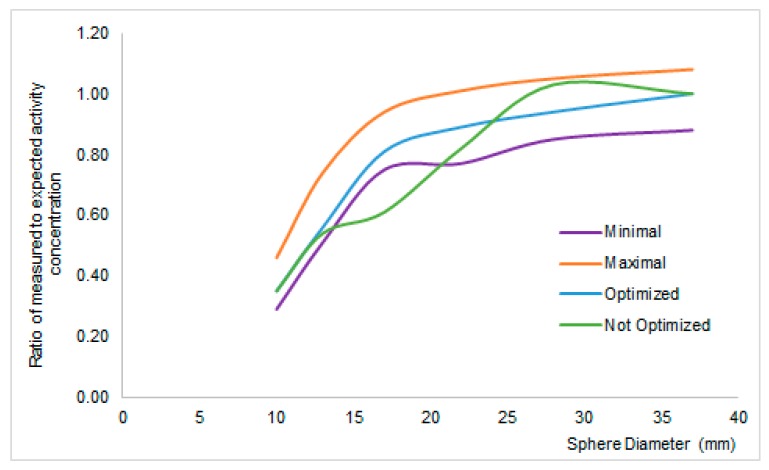
Recovery coefficient curves describing the loss of uptake in the function of a lesion’s size. The blue line is an example in which the reconstruction algorithm has been optimized in respect to the green line. Purple and orange curves are tolerated deviations.

A plethora of reconstruction algorithms exists and many differences arise when the measurements are carried out in small volumes, where loss of count due to spill-out to colder areas, the so-called partial volume effect [33], occurs. The recovery coefficient curve (Figure 2) is a figure of merit describing the ratio between real and measured activity varying the dimension of the volumes. Ideally the ratio should be one with a 10% variation due to the scanner calibration as seen above. Actually, the ratio is one for larger volumes and decreases slowly when the dimensions of the volumes are smaller than 5–8 mL [21]. A National Electrical Manufacturers Association (NEMA) phantom with hollow spheres, which could be filled with a known activity, is used to tune the reconstruction algorithm. Several parameters of the reconstruction algorithm, such as number of iterations, number of subsets, and width of Gaussian smoothing filter could hence be tuned to achieve the best recovery.

### 3.2. Site-Related Factors

These factors are related to the procedure used by each PET/CT site for the patient’s preparation and for acquisition of the images. These are influenced by the organization of the PET/CT unit and its equipment.

#### 3.2.1. Imaging Parameters

Scan duration per bed position and amount of FDG activity directly affect the image quality and quantitative results [22]. Low scan duration and low activity reduce image counts with the results of noisier images. Even though it is counterintuitive, high-injected activity can also decrease the quality of the images, since they increase the proportion of random events, which do not participate in the creation of the images. Not exceeding in very low scans per bed (i.e., below 1 min), scaling the administered activity to the patient’s weight [22] and considering increasing time instead of activity for larger patients [34] all assure a good homogenization among sites.

#### 3.2.2. Patient’s Weight

Measuring the patient’s weight with a calibrated scale is of easy implementation also in a busy department and assures the lowest variability even if, as demonstrated recently [35], patients have a very good knowledge of their weight and a variation greater than 10% only happens in 2.5% of the cases.

#### 3.2.3. Administered Activity

**Figure 3:**
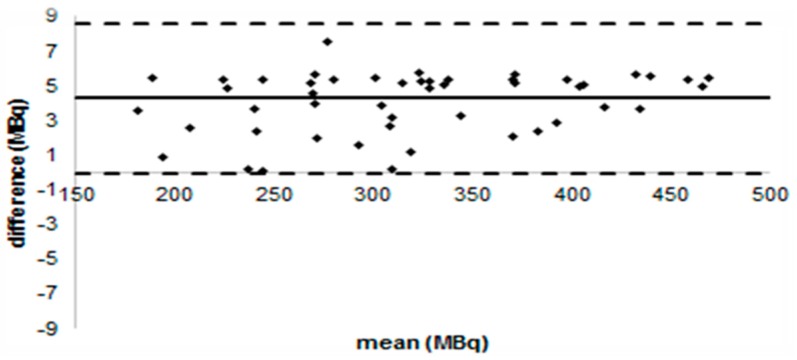
Residual activity remaining in syringe after injection versus injected activity in a cohort of 50 patients from Santa Croce e Carle Hospital. Injection is carried out with a three-way valve system and the syringe is flushed with physiological saline after the injection. Full line represents average residual activity while dashed lines are the 95% confidence interval (±2 standard deviation).

The administered activity is the difference between the activity measured in the syringe before administration and the activity measured in the syringe after the injection, often called residual activity. If injection is carried out with a three-way valve system and the syringe is flushed with physiological saline after the injection, the residual activity is less than 1% of the injected activity. As an example, Figure 3 shows the Bland-Altman plot of activity before and after administration. We can see that there is a small bias of about 3 MBq with only 5% of the difference being larger than 6 MBq.

#### 3.2.4. Other Factors

Clock synchronization should be carried out on all the clocks of the department with respect to the scanner and the dose calibrator clock to avoid biases in time and, consequently, in uptake assessments. Iodinated contrast media could modify the uptake of a lesion and are therefore not recommended for PET/CT studies if SUV is used for referral [22]. If a diagnostic CT using contrast media is performed as part of the PET/CT, a general recommendation is to perform low dose scan CT for attenuation correction before the PET scan and subsequently the full dose diagnostic CT after PET exam [22].

### 3.3. Host Factors

These factors are related to the accumulation of FDG in patients, which depends on uptake time, plasma glucose levels and patient motion or breathing artefacts.

#### 3.3.1. Uptake Time

It is a standard practice for most centres around the world to have an uptake time of 60 min, defining the uptake time as the interval between the intravenous administration of FDG and the starting of PET/CT image acquisition. Variations in uptake period are known to substantially influence measured SUV. It is generally accepted that uptake decreases in physiological organs [36] but increases in tumours. It has been postulated that imaging at longer times would improve the contrast between the tumour and normal tissue and, therefore, the ability to detect malignant lesions either at the primary or metastatic sites. However, for now, there is relative paucity of data to support this supposition.

**Figure 4:**
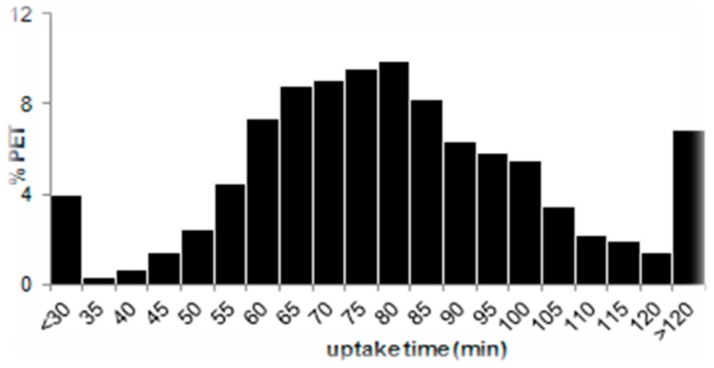
Histogram of uptake time in 1700 PET/CT scans acquired from over 56 PET sites across the world. The average uptake time is 79 ± 28 min (range 23–256).

To reduce the effect of SUV variation with uptake time, the PET/CT sites set an uptake time within a 20 min range (50–70 min) as advised in several guidelines for PET/CT procedure. Unluckily, in busy clinics, it is difficult to keep it constant and deviation occurs. The variability of uptake time, as we can see in the example of multi-centre clinical trials shown in Figure 4, is relatively high and only 30% of the PET/CT scans were acquired within this range.

**Figure 5:**
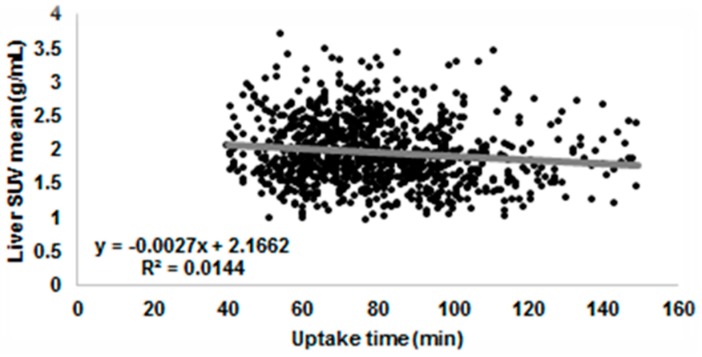
SUV of the liver as a function of uptake time in 1132 PET scans acquired from over 56 PET sites across the world. SUV of the liver is the mean SUV in a 5 cm diameter circle positioned in the VII–VIII lobes far from liver’s edge and dome. The average SUV is 1.97 ± 0.55 min (range 0.42–8.87).

**Figure 6:**
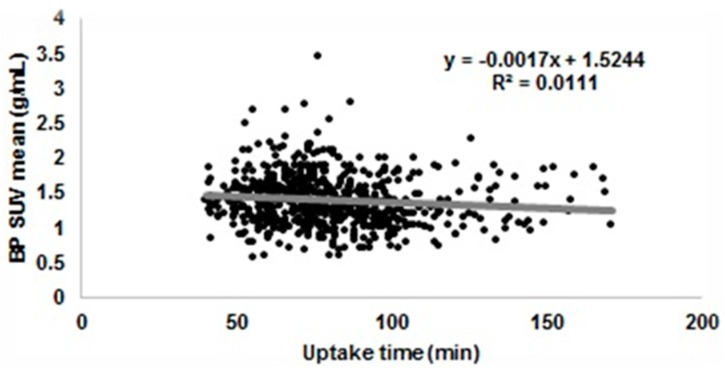
SUV of the blood pool as a function of uptake time in 1132 PET scans acquired from over 56 PET sites across the world. SUV of the blood pool is the mean SUV of a 1 cm diameter circle positioned in the descending aorta. The average SUV is 1.40 ± 0.38 min (range 0.40–3.45).

The effect of uptake time on the SUV of liver and of blood pool that are often used as internal reference is shown in Figure 5 and Figure 6. We can see how there is a general decrease of SUV (about 8% SUV in one hour), but it is lost in the general variability of SUV (about 25%).

#### 3.3.2. Glucose Level

An elevated glycaemia decreases FDG uptake caused by the tumour because normal glucose competes with FDG, leading to erroneously low SUV values [21,32]. A constant plasma glucose level in the range of 4–7 mmol/L in an individual patient across all longitudinal studies, and a track of measured values are an achievable goal with a concerted team effort [22]. No corrections to SUV based on blood glucose level have proven to be reliable.

#### 3.3.3. Extravasation

Even if is known that extravasation affects SUV, its effect is poorly reported in the literature. Some authors [37] reported 18% of occurrence of extravasation with a small fraction of patients presenting considerable extravasation up to 22% of the injected dose. Generally, a three-way valve system is preferable to syringe for tracer injection and could avoid most of extravasation. The presence of extravasation could be established if the locum of injection (i.e., the arm) is included in the axial field of view of PET scans, though no correction has, as of yet, been validated.

#### 3.3.4. Other Factors

Hydration, rest and comfort on the PET scanner table are minimal requirements to avoid uptake in muscles and patient’s motion during the scans. Breathing protocols could be used to reduce the probability of artefacts at the dome of the liver [38]. PET and CT fusion images should be visually analysed to identify possible patient motion near a lesion and, should this happen, SUV and related metrics must not be trusted.

## 4. Strategies of Error Reduction in Onco-Heamatological Clinical Trials

Many clinical trials use PET in decision-making. We browsed www.clinicaltrials.gov on 16 August 2016 with the following conditions on clinical trials: prospective, interventional, phase 2 and 3 and with the following keywords: PET and lymphoma. A total of 58 clinical trials were recruiting, and 19 had finished the enrolment in the last five years. We browsed www.pubmed.gov for the already completed studies and, excluding those that were single site and whose data were not published yet, we found 14 studies. In four of them, no information was given on the use of PET/CT and they were not considered in this review. We hence analysed the ten remaining studies and highlighted in a numbered-list fashion which of the three issues (1. the use of a central review for PET/CT assessment; 2. the equalization of PET/CT scanner; and 3. the harmonization of PET/CT procedure) was addressed and explained briefly how.

### 4.1. Hodgkin Lymphoma (HL)

#### 4.1.1. RAPID

In the RAPID trial [39] from the National Cancer Research Institute (NCRI), 602 patients with newly diagnosed stage IA and IIA HL received three cycles of ABVD (doxorubicin, bleomycin, vinblastine, and dacarbazine) and then underwent PET/CT scanning. Patients with negative PET/CT were randomly assigned to receive involved-field radiotherapy or no further treatment; patients with positive PET/CT received a fourth cycle of ABVD and radiotherapy.

(1) The images were transmitted to the core laboratory at St. Thomas’ Hospital, King’s College, London, for central review. Two experienced reporters independently scored the scans with the use of the 5-point Deauville scale to evaluate the degree of FDG uptake, if present, as well as the likelihood of residual disease. Any differences in opinion were resolved by consensus.
(2) PET scanning was performed on full-ring PET or PET/CT cameras at sites within the United Kingdom NCRI PET Research Network. Sites complied with commonly agreed methods for quality control to ensure that the performance of imaging equipment, data transfer, and image quality were within an acceptable range which was pre-specified by the core laboratory [29]. Physicists from the core laboratory visited each PET site and scanned two phantoms to check image quality and quantitative accuracy before starting the study. If the difference between expected and measured activity in the cylindrical phantom used for cross-calibration is below 10% and if the recovery coefficient of the different scanners are within ±0.25 SUV variation, the PET/CT scanner is qualified for the trial.
(3) Before undergoing scanning, patients fasted for 6 h, after which 350–400 MBq of FDG was administered intravenously. Scans were acquired 60 min later from the skull vertex or base of the brain to the upper thighs.

#### 4.1.2. RATHL

In the RATHL trial from NCRI, the Lymphoma Study Association (LYSA) and the Italian Foundation for Lymphoma (FIL), 1214 patients with newly diagnosed advanced classic HL underwent a baseline PET/CT scan, received two cycles of ABVD, and then underwent an interim PET-CT scan. Patients with negative PET/CT were randomly assigned to continue ABVD or omit bleomycin in cycles three through six. Those with positive PET/CT received BEACOPP (bleomycin, etoposide, doxorubicin, cyclophosphamide, vincristine, procarbazine, and prednisone). Radiotherapy was not recommended for patients with negative findings on interim scans.

(1) Scans were centrally reported by a network of national core laboratories in the United Kingdom, Italy, Sweden, Denmark, and Australia. Images were centrally reviewed with the use of the 5-point Deauville scale. Two readers at each core laboratory who were unaware of the patient’s clinical status scored the scans. Differences were resolved by consensus between two doctors at the same core laboratory or, when agreement could not be reached, by a third doctor at another core laboratory.
(2) No information was given about the equalization of PET/CT scanners. Patients from the UK nevertheless rely on the NCRI network for PET sites as described in the RAPID trial.
(3) Baseline PET/CT was performed within 28 days before enrolment. Interim PET/CT scanning was performed 9 to 13 days after the preceding dose of chemotherapy. Patients underwent PET/CT scanning with low-dose unenhanced PET/CT scans and were acquired at 60 ± 10 min after the intravenous injection of 350–550 MBq of FDG. Subsequent PET/CT scanning was performed under the same conditions and on the same scanner as baseline scanning.

#### 4.1.3. S0816

In S0816 trial [40] from Southwest Oncology Group (SWOG), Cancer and Leukemia Group B/Alliance (CALGB), Eastern Cooperative Oncology Group (ECOG), and the AIDS Malignancy Consortium, 358 previously untreated patients with stage III and IV HL underwent a PET/CT scan at baseline and after two initial cycles of ABVD. PET-negative patients received an additional four cycles of ABVD, whereas PET-positive patients were switched to escalated BEACOPP for six cycles.

(1) There were 331 of 358 PET/CT scans submitted for centralized review to the CALGB imaging core lab. The CALGB Imaging Core Lab enables internet-based visual and virtual conferences that allow the simultaneous display of and mutual communication between participating sites and the core lab in a secure manner. The central PET/CT review was completed in less than two days in 78% and in less than four days in 95% of the patients. The 5 reviewers scored the scans using the 5-point Deauville scale. There was one adjudicator in the CALGB Core Lab, for cases where major discrepancies existed between the local site and the central PET/CT interpretation. Scans given Deauville scores 1 to 3 were considered PET2-negative, and scans given Deauville scores 4 to 5 were considered PET-positive.
(2) Only full-ring dedicated PET/CT scanners were acceptable and older “stand-alone” PET scans were not adequate for this study. A documented daily quality control procedure had to be in place at each imaging facility. The proposed data acquisition/reconstruction protocol (including details of all the parameters above) had to be discussed with the core lab prior to the start of the study.
(3) The clinical trial protocol foresaw very detailed instruction for PET/CT scanning including patient preparation, FDG administration, uptake time in the range 60–80 min which are furnished in supplemental material along with the published article [40]. The CT of the PET/CT was used for attenuation correction of PET data and anatomic localization. CT settings followed institutional guidelines (usually 120–140 kV, at least 60 mA).

#### 4.1.4. HD15

The HD15 study [41] from German Hodgkin Study Group (GHSG) enrolled 2126 patients with advanced-stage HL from hospitals and practices in Germany, Switzerland, Austria, The Netherlands, and the Czech Republic. Six to eight cycles of BEACOPP-based chemotherapy were administered. Patients achieving partial remission after chemotherapy with at least one involved nodal site of 2.5 cm in the maximal long axis diameter underwent PET/CT scanning. PET/CT scanning by using FDG was performed two to six weeks after the end of chemotherapy to allow a prompt start of radiotherapy no later than six weeks after chemotherapy in PET-positive patients.

(1) A multidisciplinary panel consisting of a medical oncologist, a radiologist, a radiation oncologist, and a nuclear medicine physician, accompanied by a statistician, reviewed all PET/CT and CT scans as well as any available X-rays. The therapeutic decision for or against radiotherapy was based on the interpretation of the PET/CT scan and was recommended by the central review panel in consensus. Partial remission was defined by protocol as tumour volume shrinkage by 50% but continuing presence of residual tissue. A positive PET/CT scan was defined visually as a focal or diffuse uptake above the activity of mediastinal blood pool structures within residual tissue.

(2) and (3) No information was given for PET/CT scanner equalization or procedure harmonization.

#### 4.1.5. H10

The H10 [42] trial from European organization for the research and treatment of cancer (EORTC), LYSA and FIL, enrolled 1137 patients with untreated clinical stage I/II HL. Early PET/CT scan was performed after two cycles of ABVD. Patients with a negative PET scan in the experimental arm continued ABVD treatment omitting involved node radiotherapy. Patients with PET/CT-positive scans were intensified to BEACOPP escalated.

(1) Prospective central reading of the early PET scan was planned in the protocol using a real-time on-line blinded independent central review [43]. For technical reasons, centralized reviews for the LYSA group started from the initiation of the trial, and for EORTC/FIL groups, it occurred from 2008 onward. In case of absence of a timely (72 h) centralized reading, the local result of the earlier PET/CT scan was decisional for further treatment in the experimental arm. A blinded second central PET/CT review was performed retrospectively after the recommendations of the independent data monitoring committee by four experts on the scans of 52 patients with events (including patients with early PET-negative and early PET-positive scans) and 52 randomly selected patients. Out of the 104 scans, 20 could not be used for second central review for logistic or technical reasons; 84 were compared with the results of the first review. Two experts from LYSA reviewed EORTC/FIL scans and vice versa. PET/CT images were scored according to the International Harmonization Project (IHP) criteria [10].

(2) and (3) No information was given for PET/CT scanner equalization or procedure harmonization.

#### 4.1.6. BV-ABVD

The BV-ABVD [44] pilot phase II trial performed by FIL enrolled 12 untreated HL patients. Patients were administered with two cycles of brentuximab vedotin at a dose of 1.8 mg/kg followed by three or six cycles of ABVD, depending on risk group, with or without RT. The response rate after brentuximab vedotin, but before starting ABVD, was assessed by PET/CT scan. PET/CT scan was performed at baseline, after two cycles of brentuximab vedotin, and at the end of treatment.

(1) A panel consisting of three independent reviewers centrally assessed baseline and after two cycles of brentuximab vedotin PET/CT scans. The review process was coordinated through the Widen website platform [17]. Widen is a system for real-time central review which collects PET/CT images from participant sites, automatically verifies protocol violations, distributes images to reviewers, permits the online submission of the PET/CT evaluations and automatically merges them, transmitting the final results of the PET/CT reviews to the local investigators and to the clinical trial data centre. The response was assessed by adopting the 5-point scale Deauville criteria as a qualitative index and the *SUV*_max_ as a quantitative index. A response was defined as a reduction in the Deauville score or, if there was no change in it, a reduction in SUV compared to baseline.
(2) Scanners underwent clinical trial qualification for semi-quantitative analysis from the FIL core lab [30]. That is, before patients’ accrual, all PET/CT sites acquire two phantoms and sends them to the core lab. If the difference between expected and measured activity in the cylindrical phantom used for cross-calibration is below 10% and if the recovery coefficient curves are smooth and within the limits given by the European Association for Nuclear Medicine (EANM) guidelines [35], the PET/CT scanner is qualified for the trial.
(3) All FIL PET/CT-oriented protocols starting after 2011 use a shared PET/CT procedure that follows 2010 EANM guidelines [35] for patient preparation, FDG and other contrast agent administration, and PET/CT acquisition protocol.

#### 4.1.7. HD0607

An interim analysis of the HD0607 [44] clinical trial performed by FIL with 512 untreated stage IIB-IV HL patients reported the effect of PET review. Patients after two cycles of ABVD were assessed by interim PET/CT scan. Patients with positive PET scans were addressed to e-BEACOPP while those with PET-negative scans continued with another four cycles of ABVD.

(1) A panel consisting of six independent readers assessed baseline and after two cycles of ABVD PET/CT scans. The response was assessed by adopting the 5-point scale Deauville criteria, considering score 4–5 positive. The readers reviewed independently the interim PET/CT scans and inserted the review in the Widen website platform [17] that calculated automatically the majority and forwarded the results of the review to the participating site. Real-time independent review was carried out: the average and median times for diagnosis exchange were 48 h and 38 h, respectively.
(2) Scanners underwent clinical trial qualification for visual analysis from the FIL core lab [30]. That is, before patients’ accrual, all PET/CT sites acquire a cylindrical phantom and send them to the core lab. If the difference between expected and measured activity in the cylindrical phantom used for cross-calibration is below 10%, the PET/CT scanner is qualified for the trial.
(3) The participating site used a shared PET/CT procedure that followed 2003 EANM guidelines [45] for patient preparation, FDG and other contrast agent administration, and PET/CT acquisition protocol.

### 4.2. Non-Hodgkin Lymphoma

#### 4.2.1. E3404

In the E3404 trial [46] from ECOG-American College of Radiology Imaging Network (ACRIN), 100 previously untreated patients with diffuse large B-cell lymphoma (DLBCL) stage III, IV, or bulky II, were enrolled. PET/CT scan was performed after three cycles of R-CHOP (rituximab, cyclophosphamide, doxorubicin, vincristine, prednisone). PET-positive patients received four cycles of R-ICE (rituximab, ifosfamide, carboplatin, etoposide), PET-negative patients received two more cycles of R-CHOP.

(1) PET/CT scans after three cycles of R-CHOP were all submitted for central review. The interpretation took place during the fourth cycle of R-CHOP chemotherapy. Institutional results were used for six of 70 PET/CT scans because images could not be acquired for central review. The review was a binary visual interpretation, which the central reviewer based on modifications of the IHP [10], customized for this trial and deemed the “ECOG criteria”: (1) only sites of abnormality at baseline were evaluated; (2) abnormal activity required both a focal appearance and intensity greater than average liver; (3) all positive nodal sites had to have an anatomic correlate; (4) activity in bone marrow and spleen was considered abnormal only if focal and clearly discernible; (5) symmetric abnormal foci in the mediastinum and hilum were considered abnormal only if the remainder of the scan was positive; and (6) new foci were considered positive only if the remainder of the scan was positive or a new lesion was focal, very intense and associated with a lesion on CT. A dedicated publication [47] was done presenting the results of central review of this clinical trial.

(2) and (3) No information was given for PET/CT scanner equalization or procedure harmonization. ACRIN firstly developed a network for clinical trial [31]] in which its core lab verified that the difference between expected and measured activity in the cylindrical phantom, used for cross-calibration, was below 10% and it is reasonable thinking that the network was active for this trial.

#### 4.2.2. SAKK38/07

There were 138 retrospective evaluations of previously untreated patients with DLBCL with a baseline positive PET scan and a measurable lesion of at least 15 mm in the SAKK38/07 clinical trial [48] from the Swiss Oncology Society (SAKK). PET/CT scan was performed after two cycles of R-CHOP and at the end of treatment.

(1) The central review was retrospectively performed at the Nuclear Medicine Department of the University Hospital of Zürich (Zürich, Switzerland). PET scans were analysed using the Deauville 5-point scale and semi-quantitative analysis; PET was considered negative if variation in *SUV*_max_ between baseline and interim scan was >66% [49].
(2) No information was given for PET/CT scanner equalization.
(3) All patients were instructed to fast for at least 4 h before injection of 370 MBq of FDG. Blood glucose level had to be measured before injection of the radiotracer. Whole-body PET scans were performed after a standardized uptake time of 60 min. Interim PET scans were performed between day 11 and day 14 of the R-CHOP-14 cycle and between day 14 and day 28 after the last rituximab infusion (after treatment ended).

#### 4.2.3. GELTAMO-2006

In the GELTAMO-2006 [50] clinical trial from Grupo Espanol de Linfomas y Trasplante de Medula Osea (GELTAMO) conducted in 20 Spanish hospitals, 71 patients with untreated, histologically proven DLCBL or grade 3B follicular lymphoma CD20+ that had a baseline PET/CT scan positive with at least one evaluable hyper-metabolic lesion were enrolled. Patients achieving negative PET/CT after three courses of R-MegaCHOP (rituximab, cyclophosphamide, doxorubicin, vincristine, prednisone) received three additional courses, whereas PET-positive patients received two courses of R-IFE (rituximab, ifosfamide, etoposide) followed by BEAM (BCNU, etoposide, cytarabine, melphalan) and autologous stem-cell transplantation.

(1) Baseline and interim PET/CT scans were retrospectively evaluated by central review in 51/71 patients by a single nuclear medicine expert. Interim PET/CT was evaluated along baseline scan with the 5-point scale Deauville criteria—considering scores of 1, 2 or 3 as negative and scores of 4 or 5 as positive—and with semi-quantitative analysis considering PET as negative if variation in *SUV*_max_ between baseline and interim scan was >66% [49].
(2) Each patient was scanned on the same PET/CT machine for baseline and subsequent assessments.
(3) No information was given for PET/CT procedure harmonization.

#### 4.2.4. IELSG26

The IELSG26 [51] clinical trial from International Extra-Nodal Study Group, NCRI and FIL enrolled 125 patients with histopathologically proven primary mediastinal B-cell lymphoma (PMBCL) of any stage, previously untreated and eligible for intensive chemo-immunotherapy with curative intent at 21 institutions from five countries. Baseline PET/CT scans were carried out within 14 days before starting treatment. Prior treatment with corticosteroids alone for up to 1 week for the relief of local compressive symptoms was allowed. For 20 patients who required urgent treatment and for whom the PET/CT scans could not be performed before therapy started, the baseline scan was omitted after discussion with the clinical coordinators. PET/CT scans were then performed at 3 to 4 weeks after the end of the chemo-immunotherapy.

(1) CD-ROMs together with essential information on the PET/CT acquisition were sent to the core laboratory for central review. A single physician with expertise in nuclear medicine performed this after the end of treatment. Uncertain interpretations were resolved with the agreement of a second expert. The review was blinded to the clinical information. The achievement of a metabolic complete response was defined, according to the IHP criteria [10] equating score 1 or 2 on the Deauville scale. The post-chemotherapy and post-radiotherapy scans were assessed according to the Deauville scale. Diffuse uptake in the spleen or marrow on the post-chemoimmunotherapy scan is considered to be a result of chemotherapy and was not scored as active disease.
(2) PET/CT imaging was performed on full-ring integrated PET/CT systems. Baseline and response PET/CT examinations for a patient were performed in the same centre by using the same PET/CT system. Each centre was required to follow active quality control and quality assessment programs.
(3) PET and CT images were acquired in the same session. Intravenous CT contrast media were not administered before the PET study. If a diagnostic CT scan using contrast was routinely performed as part of the PET/CT examination, it was performed after the PET scan. All patients fasted for at least 6 h before the injection of 4.5 MBq/kg of FDG. Serum glucose level measured before injection of the radiotracer was less than 160 mg/dL in all patients. After a standardized uptake time of 55–65 min, PET/CT data were acquired from the mid-thigh toward the base of the skull in two-dimensional or three-dimensional mode. The PET/CT acquisition time was at least 3 min per cradle position.

#### 4.2.5. PRIMA

PET/CT after induction therapy was performed on160 patients with previously untreated high-tumour burden FL enrolled in the PRIMA [52] clinical trial from Groupe d’Etudes des Lymphomes de l’Adulte (GELA) and GOELAMS. Patients were treated with six cycles of R-CHOP or eight cycles of rituximab plus cyclophosphamide, vincristine and prednisone (R-CVP).

(1) For the retrospective central review, all participating investigators having performed PET/CT scans in the initial analysis were asked to submit on CD-ROM the PET/CT data at baseline and/or post-induction. The scans were read independently by two experienced nuclear medicine physicians. In the event of a discrepant interpretation, a third reader provided adjudication. Post-induction PET response was assessed using the IHP criteria and the Deauville 5-point scale. *SUV*_max_ was measured for each involved nodal and extranodal site.
(2) No information was given for PET/CT scanner equalization.
(3) PET/CT scanners varied among centres but for all scans data acquisition was from the skull base to the upper thighs, after fasting 4–6 h with a recorded median blood glucose of 5.3 mmol/L for baseline scans and 5.9 mmol/L for post-induction scans. Median injected FDG activity was 360 MBq (range 200–668 MBq) with a median activity per weight of 5.0 MBq/kg. The median uptake time was 60 min (range 45–110).

#### 4.2.6. PET-Folliculaire

In the PET-Folliculaire [53] clinical trial from Groupe d’Etudes des Lymphomes de l’Adulte (GELA) and GOELAMS, 121 patients with previously untreated high-tumour burden FL were treated with six cycles of R-CHOP plus two cycles of rituximab, without rituximab maintenance. PET/CT was performed before treatment, after four cycles of R-CHOP (interim PET), and at the end of treatment.

(1) PET scans were centrally reviewed by three experienced nuclear medicine physicians on a dedicated network of workstations [43]. Differences between observers were resolved by majority view. PET/CT results were reported using the Deauville 5-point scale. Two different thresholds were compared to define positivity and negativity: residual activity greater than the liver activity (scores 4 and 5), and residual activity greater than the mediastinal blood pool (scores 3, 4, and 5).
(2) Regular testing of image quality performed by a qualified physicist as recommended by the SFPM (French Society of Medical Physics) was required from each centre.
(3) PET/CT was performed in each centre on a dedicated PET/CT scanner according to standardized modalities, taking into account the technical characteristics of each camera. Patients fasted for at least 6 h before each scan and had to have a blood glucose concentration <10 mmol/L. They were administered intravenous injections of 3.5 to 8 MBq/kg (minimal activity, 185 MBq) FDG and were asked to lie in supine position for 1 h to avoid muscular uptake. Imaging was performed to cover a volume starting from the upper thigh to the skull base. Images were reconstructed iteratively with and without attenuation correction.

## 5. Evaluation of Error Reduction Strategies in Onco-Heamatological Clinical Trials

All the clinical trials analysed in the previous section recognize the need of standardizing and harmonizing PET/CT in clinical trials and address the following three issues: (1) the use of a central review for PET/CT assessment; (2) the equalization of PET/CT scanner; and (3) the harmonization of PET/CT procedure with different strategies.

### 5.1. Central Review

The use of a central review is fundamental to assess the highest reproducibility of the results and is considered binding when the primary endpoint of a clinical trial is based on tumour measurement (e.g., progression-free survival or objective response rate) [54]. The central review could be classified in several categories: independent (I) vs. consensus (C) vs. adjudicator (A), multi (M) vs. single readers (S), concentrated (C) vs. distributed (D), stand-alone (S) vs. mixed (M), and real-time (RT) vs. retrospective (R). Table 1 categorizes the central review of the analysed trials based on these categories.

#### 5.1.1. Independent versus Consensus versus Adjudicator

The readers of the images could interpret the PET/CT scans independently from the others or together in consensus. The majority of concordant results gives the overall result of the review, in the case of independent readers. In this case, the readers should be odd numbered and at least more than three. In case of consensus, there should be at least two readers and the overall result of the review is obtained by discussion among them. A third kind of review is when two reviewers analyse independently the scan and a third reviewer, un-blinded from the results, adjudicates the final result.

#### 5.1.2. Multi versus Single Readers

Single-reader review is a particular type of central review since is carried out by a single reader, usually an internationally renown expert in the field. It is used to homogenize the readings, but compared to multi-readers, it is affected by the bias of having a single reader.

#### 5.1.3. Concentrated versus Distributed

This category refers to the way in which images are exchanged for the review. In the central review, all the images are collected in a single site, usually the core lab of the clinical trial where readers sit. Investigators from participating sites upload the images to the core lab and the readers analyse them there. In the distributed review, the readers are not gathered together but could be anywhere. Hence images need to be distributed through the network to give access to remote readers. In this case, results need to be circulated again through the network and combine in the final review.

#### 5.1.4. Stand-Alone versus Mixed

Sometimes the evaluation of the local investigator in the participating site could be mixed with the one of the central review to form the final evaluation. This is usually done when not enough readers could be gathered or when the review is urgent.

#### 5.1.5. Real-Time versus Retrospective

A real-time review requires considerably more effort than a retrospective one. If PET/CT results are used for changing therapy, it is mandatory that the review results come out as soon as possible, usually within 3–5 days. In this case, all the technical issues due to PET/CT should be expeditiously resolved. An automatic system for image exchange is mandatory.

In prospective clinical trials in which PET/CT is used for patient management or it influences the primary end-point multi-readers stand-alone independent review is mandatory. Several trials demonstrated that it could be done in real-time if the result of the PET/CT affects immediately the patient’s management. Distributed review is the best alternative for reaching a higher number of independent reviewers and does not rely on a single site’s experience.

### 5.2. PET/CT Scanner Equalization

It must be noted that in multi-centre clinical trials, an SUV measurement variation across PET/CT scanners in the range of 10%–25% due to intrinsic variability of the instruments is common [55]. Hence, cross-calibration of PET/CT scanners and ancillary instrumentation is the first condition to achieve an accuracy in tracer uptake measurement to 5%–10% [28]. Several programs for the cross-calibration of PET/CT scanners have been carried out in recent years by imaging and oncology societies: the EANM (EANM) accreditation program for site of excellence carried out by EARL Ltd. (Wien, Austria) [23,56], the UK NCRI PET Clinical Trial Network [29], the ACRIN program [31], the Clinical Trial Network (CTN) of Society of Nuclear Medicine and Molecular Imaging (SNMMI) [32,57], the JSCT NHL10 trial [58] in Japan and the FIL Core lab in Italy [30]. While these programs are common nowadays, at the time the clinical trial discussed in this manuscript started, only the RAPID, the S0186 and the BV-ABVD clinical trials addressed the problem of standardization of scanners through a thorough clinical trial qualification process consisting of the verification that the requirements for PET/CT scanners were fulfilled. Others, such as the RATHL, the PET-Folliculaire and the IELSG26, only required documentation from the local centre assessing that they were fulfilling the national or international standards for quality assurance.

### 5.3. Harmonization of PET Procedure

Despite of the publication of guidelines for scanning patients [35,59], the lack of standardization [60,61] has hampered in the past the use of SUV as a biomarker in clinical trials. Now, thanks to better knowledge of the factors affecting SUV measurements [26], guidelines for patient scanning and PET/CT image acquisition [62] are recommended to improve data quality and reproducibility [62,63]. All the studies prescribed correct timing for PET/CT scanning in respect to concurrent therapy in order to avoid the effects of medicaments on PET/CT studies (i.e., steroids). RAPID, S0186, IELSG26 and PET-Folliculaire adopted international guidelines to be followed and report the parameters used in the trials.

## 6. Conclusions

Major clinical trials that use positron emission tomography computed tomography (PET/CT) as a management tool use a thorough and careful program to achieve the reproducibility of the PET/CT data. As we saw, most of the trials used PET in a qualitative way, and therefore some of the requirements for clinical trial qualification are more relaxed in respect to a quantitative approach. Several new clinical trials that are running foresee use of PET/CT-derived standardized uptake value (SUV) metrics and are hence using a severer program for trial qualification that permits lower variability among scanners below 5% and use an acceptance/rejection schema for low standardized PET/CT scans, which have some factors, such as uptake time, that are outside predefined limits.

## Figures and Tables

**Table 1:** Categories of central review used by the clinical trials analysed in this manuscript.

Clinical Trials	Independent vs. Consensus vs. Adjudicator	Multi vs. Single Readers	Concentrated vs. Distributed	Stand-Alone vs. Mixed	Real-Time vs. Retrospective
RAPID	A	M	C	S	RT
RATHL	A	M	C	S	RT
S0816	A	M	D	M	RT
HD15	C	M	C	S	RT
H10	-	M	D	M	RT
BV-ABVD	I	M	D	S	R
HD0607	I	M	D	M	RT
E3404	I	M	D	S	RT
SAKK38/07	I	S	C	S	R
GELTAMO-2006	-	S	C	S	R
IELSG26	-	S	C	S	R
PRIMA	A	M	C	M	R
PET-Folliculaire	I	M	D	S	R

## References

[1] Jerusalem G., Hustinx R., Beguin Y., Fillet G. (2002). The value of positron emission tomography (PET) imaging in disease staging and therapy assessment. Ann. Oncol..

[2] Gallamini A., Zwarthoed C., Borra A. (2014). Positron Emission Tomography (PET) in Oncology. Cancers.

[3] Kostakoglu L., Cheson B.D. (2014). Current role of FDG PET/CT in lymphoma. Eur. J. Nucl. Med. Mol. Imaging.

[4] Biggi A., Guerra L., Hofman M.S. (2015). Current status of FDG-PET/CT in staging of adult lymphoma. Clin. Transl. Imaging.

[5] Cheson B., Fisher R., Barrington S. (2014). Recommendations for initial evaluation, staging, and response assessment of Hodgkin and Non-Hodgkin lymphoma: The Lugano classification. J. Clin. Oncol..

[6] Hutchings M., Barrington S.F. (2009). PET/CT for therapy response assessment in lymphoma. J. Nucl. Med..

[7] Hutchings M., Barrington S. (2015). FDG-PET for the early treatment monitoring, for final response and follow-up evaluation in lymphoma. Clin. Transl. Imaging.

[8] Meignan M., Gallamini A., Haioun C. (2009). Report on the First International Workshop on Interim-PET-Scan in Lymphoma. Leuk. Lymphoma.

[9] Wahl R.L., Jacene H., Kasamon Y., Lodge M.A. (2009). From RECIST to PERCIST: Evolving Considerations for PET response criteria in solid tumors. J. Nucl. Med..

[10] Juweid M.E., Stroobants S., Hoekstra O.S., Mottaghy F.M., Dietlein M., Guermazi A., Wiseman G.A., Kostakoglu L., Scheidhauer K., Buck A. (2007). Use of Positron Emission Tomography for Response Assessment of Lymphoma: Consensus of the Imaging Subcommittee of International Harmonization Project in Lymphoma. J. Clin. Oncol..

[11] Meignan M., Itti E., Gallamini A. (2015). FDG PET/CT imaging as a biomarker in lymphoma. Eur. J. Nucl. Med. Mol. Imaging.

[12] Kostakoglu L., Chauvie S. (2015). PET-derived metabolic volume metrics in lymphoma. Clin. Transl. Imaging.

[13] Gallagher B.M., Fowler J.S., Gutterson N.I., Macgregor R.R., Wolf A.P. (1978). Metabolic Trapping as a Principle of Radiopharmaceuticals Design: Some Factors Responsible for the Biodistribution of FDG. J. Nucl. Med..

[14] Laffon E., Adhoute X., de Clermont H., Marthan R. (2011). Is Liver SUV Stable over Time in 18F-FDG PET Imaging?. J. Nucl. Med. Technol..

[15] Barrington S., Qian W., Somer E., Franceschetto A., Bagni B., Brun E., Almquist A., Højgaard L., Federico M., Gallamini A. (2010). O’Doherty Concordance between four European centres of PET reporting criteria designed for use in multicentre trials in Hodgkin lymphoma. Eur. J. Nucl. Med. Mol. Imaging.

[16] Biggi A., Gallamini A., Chauvie S., Hutchings M., Kostakoglu L., Gregianin M., Meignan M., Malkowski B., Hofman M.S., Barrington S.F. (2013). International validation study for interim PET in ABVD-treated, advanced-stage hodgkin lymphoma: Interpretation criteria and concordance rate among reviewers. J. Nucl. Med..

[17] Chauvie S., Biggi A., Stancu A., Cerello P., Cavallo A., Fallanca F., Ficola U., Gregianin M., Guerra U.P., Chiaravalloti A. (2014). WIDEN: A tool for medical image management in multicenter clinical trials. Clin. Trials.

[18] Itti E., Meignan M., Berriolo-Riedinger A., Biggi A., Cashen A.F., Véra P., Tilly H., Siegel B.A., Gallamini A., Casasnovas R.-O. (2013). An international confirmatory study of the prognostic value of early PET/CT in diffuse large B-cell lymphoma: Comparison between Deauville criteria and ΔSUVmax. Eur. J. Nucl. Med. Mol. Imaging.

[19] Ceriani L., Barrington S., Biggi A., Malkowski B., Metser U., Versari A., Martelli M., Davies A., Johnson P.W., Zucca E. (2016). Training improves the interobserver agreement of the expert positron emission tomography review panel in primary mediastinal B-cell lymphoma: Interim analysis in the ongoing International Extranodal Lymphoma Study Group-37 study. Hematol. Oncol..

[20] Kalpadakis C., Pangalis G.A., Dimopoulou M.N., Vassilakopoulos T.P., Kyrtsonis M.-C., Korkolopoulou P., Kontopidou F.N., Siakantaris M.P., Dimitriadou E.M., Kokoris S.I. (2007). Rituximab monotherapy is highly effective in splenic marginal zone lymphoma. Hematol. Oncol..

[21] Boellaard R., O’Doherty M.J., Weber W.A., Mottaghy F.M., Lonsdale M.N., Stroobants S.G., Oyen W.J.G., Kotzerke J., Hoekstra O.S., Pruim J. (2010). FDG PET and PET/CT: EANM procedure guidelines for tumour PET imaging: Version 1.0. Eur. J. Nucl. Med. Mol. Imaging.

[22] Boellaard R., Delgado-Bolton R., Oyen W.J.G., Giammarile F., Tatsch K., Eschner W., Verzijlbergen F.J., Barrington S.F., Pike L.C., Weber W.A. (2015). European Association of Nuclear Medicine (EANM) FDG PET/CT: EANM procedure guidelines for tumour imaging: Version 2.0. Eur. J. Nucl. Med. Mol. Imaging.

[23] Zijlstra J.M., Boellaard R., Hoekstra O.S. (2009). Interim positron emission tomography scan in multi-center studies: Optimization of visual and quantitative assessments. Leuk. Lymphoma.

[24] Makris N.E., Huisman M.C., Kinahan P.E., Lammertsma A.A., Boellaard R. (2013). Evaluation of strategies towards harmonization of FDG PET/CT studies in multicentre trials: Comparison of scanner validation phantoms and data analysis procedures. Eur. J. Nucl. Med. Mol. Imaging.

[25] Lammertsma A.A., Boellaard R. (2014). The need for quantitative PET in multicentre studies. Clin. Transl. Imaging.

[26] Adams M.C., Turkington T.G., Wilson J.M., Wong T.Z. (2010). A systematic review of the factors affecting accuracy of SUV measurements. Am. J. Roentgenol..

[27] Boellaard R. (2011). Methodological aspects of multicenter studies with quantitative PET. Methods Mol. Biol..

[28] Geworski L., Knoop B.O., de Wit M., Ivancević V., Bares R., Munz D.L. (2002). Multicenter comparison of calibration and cross calibration of PET scanners. J. Nucl. Med..

[29] Barrington S.F., Mackewn J.E., Schleyer P., Marsden P.K., Mikhaeel N.G., Qian W., Mouncey P., Patrick P., Popova B., Johnson P. (2011). Establishment of a UK-wide network to facilitate the acquisition of quality assured FDG-PET data for clinical trials in lymphoma. Ann. Oncol..

[30] Chauvie S. (2016). The ^68^Ge phantom-based FDG-PET site qualification program for clinical trials adopted by FIL (Italian Foundation on Lymphoma). Phys. Med..

[31] Scheuermann J.S., Saffer J.R., Karp J.S., Levering A.M., Siegel B.A. (2009). Qualification of PET scanners for use in multicenter cancer clinical trials: The American College of Radiology Imaging Network experience. J. Nucl. Med..

[32] Christian P. (2012). Use of a precision fillable clinical simulator phantom for PET/CT scanner validation in multi-center clinical trials: The SNM Clinical Trials Network (CTN) Program. J. Nucl. Med..

[33] Soret M., Bacharach S.L., Buvat I. (2007). Partial-volume effect in PET tumor imaging. J. Nucl. Med..

[34] Matheoud R., Della Monica P., Secco C., Loi G., Krengli M., Inglese E., Brambilla M. (2011). Influence of different contributions of scatter and attenuation on the threshold values in contrast-based algorithms for volume segmentation. Phys. Med..

[35] Lasnon C., Houdu B., Kammerer E., Salomon T., Devreese J., Lebasnier A., Aide N. (2016). Patient’s weight: A neglected cause of variability in SUV measurements? A survey from an EARL accredited PET centre in 513 patients. Eur. J. Nucl. Med. Mol. Imaging.

[36] Weber W.A., Ziegler S.I., Thödtmann R., Hanauske A.R., Schwaiger M. (1999). Reproducibility of metabolic measurements in malignant tumors using FDG PET. J. Nucl. Med..

[37] Silva-rodríguez J., Aguiar P., Sánchez M., Mosquera J., Luna-vega V., Cortés J., Garrido M., Pombar M., Ruibal Á. (2014). Correction for FDG PET dose extravasations: Monte Carlo validation and quantitative evaluation of patient studies Correction for FDG PET dose extravasations: Monte Carlo validation. Med. Phys..

[38] Graham M.M., Wahl R.L., Hoffman J.M., Yap J.T., Sunderland J.J., Boellaard R., Perlman E.S., Kinahan P.E., Christian P.E., Hoekstra O.S. (2015). Summary of the UPICT Protocol for 18F-FDG PET/CT Imaging in Oncology Clinical Trials. J. Nucl. Med..

[39] Radford J., Illidge T., Counsell N., Hancock B., Pettengell R., Johnson P., Wimperis J., Culligan D., Popova B., Smith P. (2015). Results of a Trial of PET-Directed Therapy for Early-Stage Hodgkin’s Lymphoma. N. Engl. J. Med..

[40] Press O.W., Li H., Sch H., Straus D.J., Moskowitz C.H., Leblanc M., Rimsza L.M., Bartlett N.L., Evens A.M., Mittra E.S. (2016). US Intergroup Trial of Response-Adapted Therapy for Stage III to IV Hodgkin Lymphoma Using Early Interim Fluorodeoxyglucose-Positron Emission Tomography Imaging: Southwest Oncology Group S0816. J. Clin. Oncol..

[41] Kobe C., Kuhnert G., Kahraman D., Haverkamp H., Eich H.T., Franke M., Persigehl T., Klutmann S., Amthauer H., Bockisch A. (2014). Assessment of tumor size reduction improves outcome prediction of positron emission tomography/computed tomography after chemotherapy in advanced-stage Hodgkin lymphoma. J. Clin. Oncol..

[42] Raemaekers J.M.M., André M.P.E., Federico M., Girinsky T., Oumedaly R., Brusamolino E., Brice P., Fermé C., Van Der Maazen R., Gotti M. (2014). Omitting Radiotherapy in early positron emission tomography-negative stage I/II Hodgkin lymphoma is associated with an increased risk of early relapse: Clinical results of the preplanned interim analysis of the randomized EORTC/LYSA/FIL H10 trial. J. Clin. Oncol..

[43] Meignan M., Itti E., Bardet S., Al E. (2009). Development and application of a real-time on-line blinded independent central review of interim PET scans to determine treatment allocation in lymphoma trials. J. Clin. Oncol..

[44] Federico M., Luminari S., Pellegrini C., Merli F., Pesce E.A., Chauvie S., Gandolfi L., Capodanno I., Salati M., Argnani L. (2015). Brentuximab vedotin followed by ABVD +/− radiotherapy in patients with previously untreated Hodgkin lymphoma: Final results of a pilot phase II study. Haematologica.

[45] Bombardieri E., Aktolun C., Baum R.P., Bishof-Delaloye A., Buscombe J., Chatal J.F., Maffioli L., Moncayo R., Mortelmans L., Reske S.N. (2003). FDG-PET: Procedure guidelines for tumour imaging. Eur. J. Nucl. Med. Mol. Imaging.

[46] Gurung P., Lukens J.R., Kanneganti T. (2016). HHS Public Access. Br. J. Haematol..

[47] Horning S.J., Juweid M.E., Schöder H., Wiseman G., Mcmillan A., Lode J., Advani R., Gascoyne R., Quon A., Horning S.J. (2010). Interim positron emission tomography scans in diffuse large B-cell lymphoma: An independent expert nuclear medicine evaluation of the Eastern Cooperative Oncology Group E3404 study CME article Interim positron emission tomography scans in diffuse large B. Blood.

[48] Mamot C., Klingbiel D., Hitz F., Renner C., Pabst T., Driessen C., Mey U., Pless M., Bargetzi M., Krasniqi F. (2015). Final Results of a Prospective Evaluation of the Predictive Value of Interim Positron Emission Tomography in Patients with Diffuse Large B-Cell Lymphoma Treated With R-CHOP-14 (SAKK 38/07). J. Clin. Oncol..

[49] Lin C., Itti E., Haioun C., Petegnief Y., Luciani A., Dupuis J., Paone G., Talbot J.-N., Rahmouni A., Meignan M. (2007). Early 18F-FDG PET for prediction of prognosis in patients with diffuse large B-cell lymphoma: SUV-based assessment versus visual analysis. J. Nucl. Med..

[50] Pardal E., Coronado M., Martín A., Grande C., Marín-Niebla A., Panizo C., Bello J.L., Conde E., Hernández M.T., Arranz R. (2014). Intensification treatment based on early FDG-PET in patients with high-risk diffuse large B-cell lymphoma: A phase II GELTAMO trial. Br. J. Haematol..

[51] Martelli M., Ceriani L., Zucca E., Zinzani P.L., Ferreri A.J.M., Vitolo U., Stelitano C., Brusamolino E., Cabras M.G., Rigacci L. (2014). [18F]fluorodeoxyglucose positron emission tomography predicts survival after chemoimmunotherapy for primary mediastinal large B-cell lymphoma: Results of the International Extranodal Lymphoma Study Group IELSG-26 study. J. Clin. Oncol..

[52] Tychyj-Pinel C., Ricard F., Fulham M., Fournier M., Meignan M., Lamy T., Vera P., Salles G., Trotman J. (2014). PET/CT assessment in follicular lymphoma using standardized criteria: Central review in the PRIMA study. Eur. J. Nucl. Med. Mol. Imaging.

[53] Dupuis J., Berriolo-Riedinger A., Julian A., Brice P., Tychyj-Pinel C., Tilly H., Mounier N., Gallamini A., Feugier P., Soubeyran P. (2012). Impact of [^18^F]fluorodeoxyglucose positron emission tomography response evaluation in patients with high-tumor burden follicular lymphoma treated with immunochemotherapy: A prospective study from the Groupe d’Etudes des Lymphomes de l’Adulte and GOELAMS. J. Clin. Oncol..

[54] US Food and Drug Administration (2007). Guidance for Industry: Clinical Trial Endpoints for the Approval of Cancer Drugs and Biologics.

[55] Fahey F.H., Kinahan P.E., Doot R.K., Kocak M., Thurston H., Poussaint T.Y. (2010). Variability in PET quantitation within a multicenter consortium. Med. Phys..

[56] Boellaard R., Hristova I., Ettinger S., Sera T., Stroobants S., Chiti A., Bauer A., Tatsch K., Verzijlbergen F., Oyen W. (2013). EARL FDG-PET/CT accreditation program: Feasibility, overview and results of first 55 successfully accredited sites. J. Nucl. Med..

[57] Sunderland J.J., Christian P.E. (2015). Quantitative PET/CT Scanner Performance Chracterization Based upon the SNMMI Clinical Trial Networ Oncology Clinical Simulator Phantom. J. Nucl. Med..

[58] Daisaki H., Tateishi U., Terauchi T., Tatsumi M., Suzuki K., Shimada N., Nishida H., Numata A., Kato K., Akashi K. (2013). Standardization of image quality across multiple centers by optimization of acquisition and reconstruction parameters with interim FDG-PET/CT for evaluating diffuse large B cell lymphoma. Ann. Nucl. Med..

[59] Delbeke D., Coleman R.E., Guiberteau M.J., Brown M.L., Royal H.D., Siegel B.A., Townsend D.W., Berland L.L., Parker J.A., Hubner K. (2006). Procedure guideline for tumor imaging with 18F-FDG PET/CT 1.0. J. Nucl. Med..

[60] Graham M.M., Badawi R.D., Wahl R.L. (2011). Variations in PET/CT methodology for oncologic imaging at U.S. academic medical centers: An imaging response assessment team survey. J. Nucl. Med..

[61] Beyer T., Czernin J., Freudenberg L.S. (2011). Variations in clinical PET/CT operations: Results of an international survey of active PET/CT users. J. Nucl. Med..

[62] Boellaard R., Oyen W.J.G., Hoekstra C.J., Hoekstra O.S., Visser E.P., Willemsen A.T., Arends B., Verzijlbergen F.J., Zijlstra J., Paans A.M. (2008). The Netherlands protocol for standardisation and quantification of FDG whole body PET studies in multi-centre trials. Eur. J. Nucl. Med. Mol. Imaging.

[63] Boellaard R. (2009). Standards for PET image acquisition and quantitative data analysis. J. Nucl. Med..

